# 

**Published:** 1879-05

**Authors:** 


MAY, 1879.

### THE AMERICAN JOURNAL OF DENTAL SCIENCE,

EDITED BY

F. J. S. GORGAS, M. D., D. D. S.,

AND

JAMES B. HODGKIN, D. D. S.

PUBLISHED MONTHLY.

$2.50 PER ANNUM.

VOL. 13.—THIRD SERIES.—No. 1.

BALTIMORE:
SNOWDEN & COWMAN.

LONDON:

TRUBNER & CO., 60 PATERNOSTER ROW.

1879.

PAYABLE IN ADVANCE.

